# Green ratio-manipulating spectrophotometric methods for simultaneous determination of empagliflozin and sitagliptin in pharmaceutical formulations

**DOI:** 10.1186/s13065-026-01935-6

**Published:** 2026-10-01

**Authors:** Ahmad A. A. Mohamad, Ahmed A. Almrasy, Ahmed H. Abdelazim, Ahmed S. Taha

**Affiliations:** Pharmaceutical Analytical Chemistry Department, Faculty of Pharmacy, Al-Azhar University, Cairo, 11751 Egypt

**Keywords:** Empagliflozin, Sitagliptin, Spectrophotometry, Ratio difference, First derivative, Green analytical chemistry

## Abstract

**Background:**

Empagliflozin (EMP) and sitagliptin (SIT) are antidiabetic agents with complementary mechanisms of action and are frequently formulated as fixed-dose combinations. However, their substantially overlapping ultraviolet absorption spectra limit the applicability of direct spectrophotometric determination in combined dosage forms.

**Objective:**

This study aimed to develop and validate simple, rapid, and low-solvent-consumption ratio-manipulating spectrophotometric methods for simultaneous determination of EMP and SIT in combined pharmaceutical formulations, and to assess their analytical, environmental, and practical performance.

**Methods:**

Two mathematical spectrophotometric approaches, ratio difference (RD) and first-derivative ratio spectrophotometry (1DD), were developed. In the RD method, EMP was quantified using the amplitude difference between 230 and 285 nm, whereas SIT was determined from the amplitude difference between 257 and 209 nm. In the 1DD method, the first derivative of the ratio spectra was measured at 239 nm for EMP and 250 nm for SIT, using Δλ = 8 nm and a scaling factor of 80. The methods were validated for linearity, accuracy, precision, selectivity, robustness, and sensitivity. Their environmental and practical profiles were assessed using the Carbon Footprint Reduction Index (CaFRI), Complex Modified Green Analytical Procedure Index (ComplexMoGAPI), Click Analytical Chemistry Index (CACI), Blue Applicability Grade Index (BAGI), and Whiteness Evaluation for Chemical Analysis (WECA) tools.

**Results:**

Both methods were linear over concentration ranges of 2–25 µg/mL for EMP and 5–100 µg/mL for SIT. For the RD method, the LOD values were 0.326 and 0.419 µg/mL, and the LOQ values were 0.978 and 1.257 µg/mL for EMP and SIT, respectively. For the 1DD method, the corresponding LOD values were 0.324 and 0.423 µg/mL, while LOQ values were 0.972 and 1.269 µg/mL for EMP and SIT, respectively. Mean recoveries ranged from 99.18% to 100.11%, with repeatability and intermediate-precision %RSD values not exceeding 0.761% and 0.979%, respectively. The proposed methods were successfully applied to commercial combined tablets, using ethanol as the analytical solvent. Student’s t- and F-tests revealed no significant difference between the proposed and reported methods at the 95% confidence level.

**Conclusion:**

The developed RD and 1DD methods provide rapid, separation-free, and reliable alternatives for simultaneous spectrophotometric determination of EMP and SIT. Their use of ethanol, minimal sample preparation, low solvent consumption, and favorable analytical, environmental, and practical profiles support their suitability for routine pharmaceutical quality-control analysis, particularly in laboratories with limited chromatographic resources.

## Introduction

**Fig. 1:**
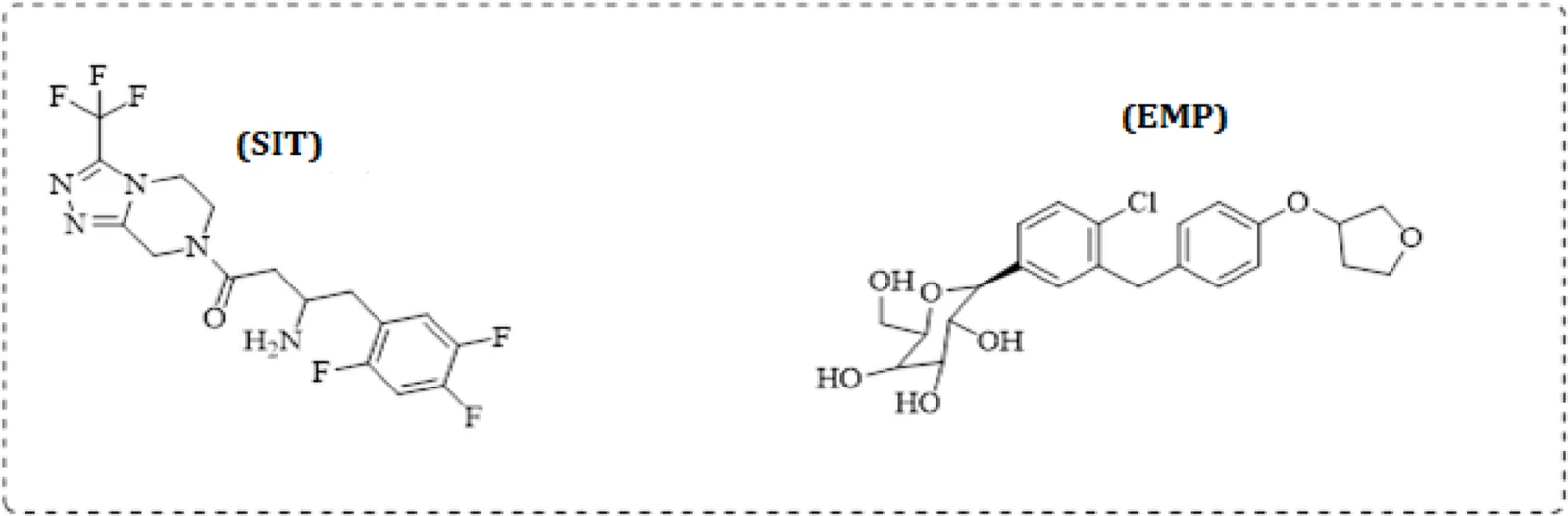
Structural formula for SIT and EMP

Type 2 diabetes mellitus is a major chronic metabolic disorder requiring sustained pharmacological treatment to achieve adequate glycemic control and reduce the risk of long-term complications [1–8]. Combination therapy with agents acting through complementary mechanisms has become an important therapeutic strategy when monotherapy provides insufficient glycemic control [9]. Empagliflozin (EMP), a sodium–glucose co-transporter 2 (SGLT2) inhibitor, reduces blood glucose levels by inhibiting renal glucose reabsorption and enhancing urinary glucose excretion [10–12]. Sitagliptin (SIT), a dipeptidyl peptidase-4 (DPP-4) inhibitor, enhances incretin activity, thereby promoting glucose-dependent insulin secretion and suppressing glucagon release [13, 14] (Fig. 1). The complementary pharmacological actions of EMP and SIT have led to their incorporation into fixed-dose combination formulations for managing type 2 diabetes mellitus [15].

The increasing use of EMP/SIT fixed-dose formulations requires reliable analytical procedures that are accurate, selective, and practical for routine pharmaceutical quality control. Several analytical techniques have been reported for determining EMP, including UV spectrophotometry [16, 17], fluorimetry [18–25], high-performance liquid chromatography (HPLC) [26–33], and electrochemical methods [34]. Similarly, SIT has been determined using various techniques, including HPLC [35–39], UV spectrophotometry [40–44], fluorimetry [45–49], and electrochemical methods [50–52]. In contrast, relatively limited attention has been devoted to their simultaneous determination in combined pharmaceutical formulations. Reported methods for the EMP/SIT combination include simultaneous-equation UV spectrophotometry [53], and reversed-phase high-performance liquid chromatography (RP-HPLC) [54]. Simultaneous-equation spectrophotometry offers simplicity, rapid analysis, and low operational requirements; however, extensive spectral overlap and dependence on accurately established absorptivity coefficients can affect performance [55].

RP-HPLC provides effective chromatographic resolution and high selectivity, but its separation-based workflow requires more instrumentation and operational effort and relies on routine mobile-phase solvents [56]. Consequently, an analytical approach that combines the simplicity of direct spectrophotometry with mathematical resolution of spectral interference remains of practical interest, particularly for routine quality-control laboratories where simplicity, reduced sample handling, and efficient resource utilization are desirable.

The zero-order absorption spectra of EMP and SIT overlap substantially across the analytical region, limiting their direct simultaneous determination by conventional spectrophotometry. Ratio-spectrum manipulation provides an established mathematical strategy to overcome this overlap without prior physical or chromatographic separation. In this approach, the spectrum of a binary mixture is divided by the spectrum of one component selected as a divisor, converting the divisor contribution into a constant term in the resulting ratio spectrum. This transformation permits selective quantification of the other component through appropriate mathematical processing. Ratio-difference (RD) spectrophotometry exploits the difference between ratio amplitudes measured at appropriately selected wavelengths to eliminate the constant divisor contribution. First-derivative ratio (1DD) spectrophotometry further processes the ratio spectrum by derivative transformation, enhancing spectral resolution and enabling selective measurement of derivative responses. However, the utility of these techniques depends closely on the spectral behavior of the analyte pair and on the appropriate selection of divisor concentration, analytical wavelengths, and processing conditions.

Accordingly, this study develops complementary RD and 1DD spectrophotometric procedures adapted to the EMP/SIT fixed-dose combination [57–61]. The methodological approach addresses the pronounced spectral overlap between the two drugs while retaining the inherent advantages of UV–visible spectrophotometry, including straightforward operation, minimal sample manipulation, and avoidance of chromatographic separation. Ethanol was selected as the analytical medium to support a more environmentally favorable workflow and to reduce dependence on less desirable organic solvents. The developed procedures were validated in accordance with ICH guidelines and applied to a commercial EMP/SIT formulation to establish their analytical reliability and practical suitability for routine pharmaceutical quality control [62]. Notably, the proposed procedures extend the analytical working ranges toward lower concentrations than those reported for the previously described simultaneous-equation spectrophotometric method. In addition, their environmental and operational profiles were evaluated using complementary sustainability-oriented assessment tools, providing a broader characterization of the proposed procedures with respect to analytical performance, practical applicability, and environmental compatibility [63–69].

## Experimental

### Materials and reagents

Empagliflozine reference standards with a stated purity of 99.89% was supplied by DPK Pharmaceuticals, Al Obour City, Egypt. Sitagliptin SIT reference standards with a stated purity of 99.51% was obtained from Hikma Pharmaceuticals Co., 6th of October City, Giza, Egypt. Gliempaflozin^®^ SITA 25/100^®^ tablets, labeled to contain 25 mg of EMP and 100 mg of SIT per tablet (batch no. EMV250616C), were manufactured by Steris Healthcare, India, and obtained from the local market in India. Analytical-grade ethanol (Sigma-Aldrich, Germany) was used as the solvent throughout the analytical procedures.

### Apparatus

Absorption measurements were performed using a Shimadzu UV-1800 UV–visible spectrophotometer (Shimadzu Corporation, Japan) equipped with 10-mm quartz cells. Data acquisition and spectral processing were performed using UV-Probe software version 2.4.

### Standards stock solutions

Separate stock solutions of EMP and SIT (1000 µg/mL) were prepared in ethanol. Appropriate quantities of each drug were accurately weighed and transferred to separate 100-mL volumetric flasks, dissolved in ethanol, and diluted to volume with the same solvent.

Working solutions were prepared by diluting the corresponding stock solutions with ethanol. Ethanol was used as the spectrophotometric blank throughout the study.

### Linearity calibration graphs

Calibration solutions were prepared over the concentration ranges of 2–25 µg/mL for EMP and 5–100 µg/mL for SIT by diluting the respective stock solutions with ethanol. For ratio-spectrum manipulation, the absorption spectrum of each analyte was divided by the spectrum of the other analyte serving as the divisor.

An SIT solution of 80 µg/mL was selected as the divisor for generation of EMP ratio spectra, whereas an EMP solution of 18 µg/mL was used as the divisor for generation of SIT ratio spectra. These divisor concentrations were selected after examining several concentrations to obtain smooth ratio spectra with satisfactory sensitivity and minimal spectral noise.

### Ratio difference method

For the determination of EMP, the difference between the amplitudes of the ratio spectra at 230 and 285 nm was measured. For SIT, the corresponding amplitude difference was calculated at 257 and 209 nm. Calibration curves were constructed by plotting the measured amplitude differences against the corresponding analyte concentrations. Linear regression analysis was subsequently used for quantitative determination.

### First-derivative ratio spectrophotometry

The ratio spectra were subjected to first-order derivative transformation using a wavelength interval (Δλ) of 8 nm and a scaling factor of 80. EMP and SIT were quantified by measuring the derivative amplitudes at 239 and 250 nm, respectively. Calibration curves were constructed by plotting the derivative amplitudes against the corresponding analyte concentrations.

### Synthetic mixtures analysis

Five synthetic mixtures containing EMP and SIT at concentration ratios corresponding approximately to their labeled ratio in the commercial formulation (1:4) were prepared over the investigated concentration ranges. Each mixture was analyzed using both the RD and 1DD procedures according to the calibration procedures described above. The calculated concentrations were compared with the nominal concentrations to assess the applicability of the proposed methods to mixtures containing both analytes.

### Pharmaceutical tablets application

Ten tablets were accurately weighed, finely powdered, and thoroughly mixed to ensure sample homogeneity. An accurately weighed portion of the powder equivalent to the average weight of one tablet (350 mg) was transferred into a 100-mL volumetric flask containing approximately 50 mL of ethanol. The mixture was sonicated for 20 min with intermittent vigorous shaking to ensure complete extraction of the active pharmaceutical ingredients. The resulting extract was first passed through Whatman filter paper to remove insoluble excipients and subsequently filtered through a 0.45-µm membrane filter. The filtrate was quantitatively transferred to the volumetric flask, and the volume was made up to the mark with ethanol. Appropriate aliquots of the resulting stock solution were further diluted with ethanol to obtain solutions within the respective working concentration ranges of the proposed methods. The prepared solutions were subsequently analyzed according to the proposed RD and 1DD spectrophotometric procedures.

## Results and discussion

### Spectral characteristics and method development

**Fig. 2:**
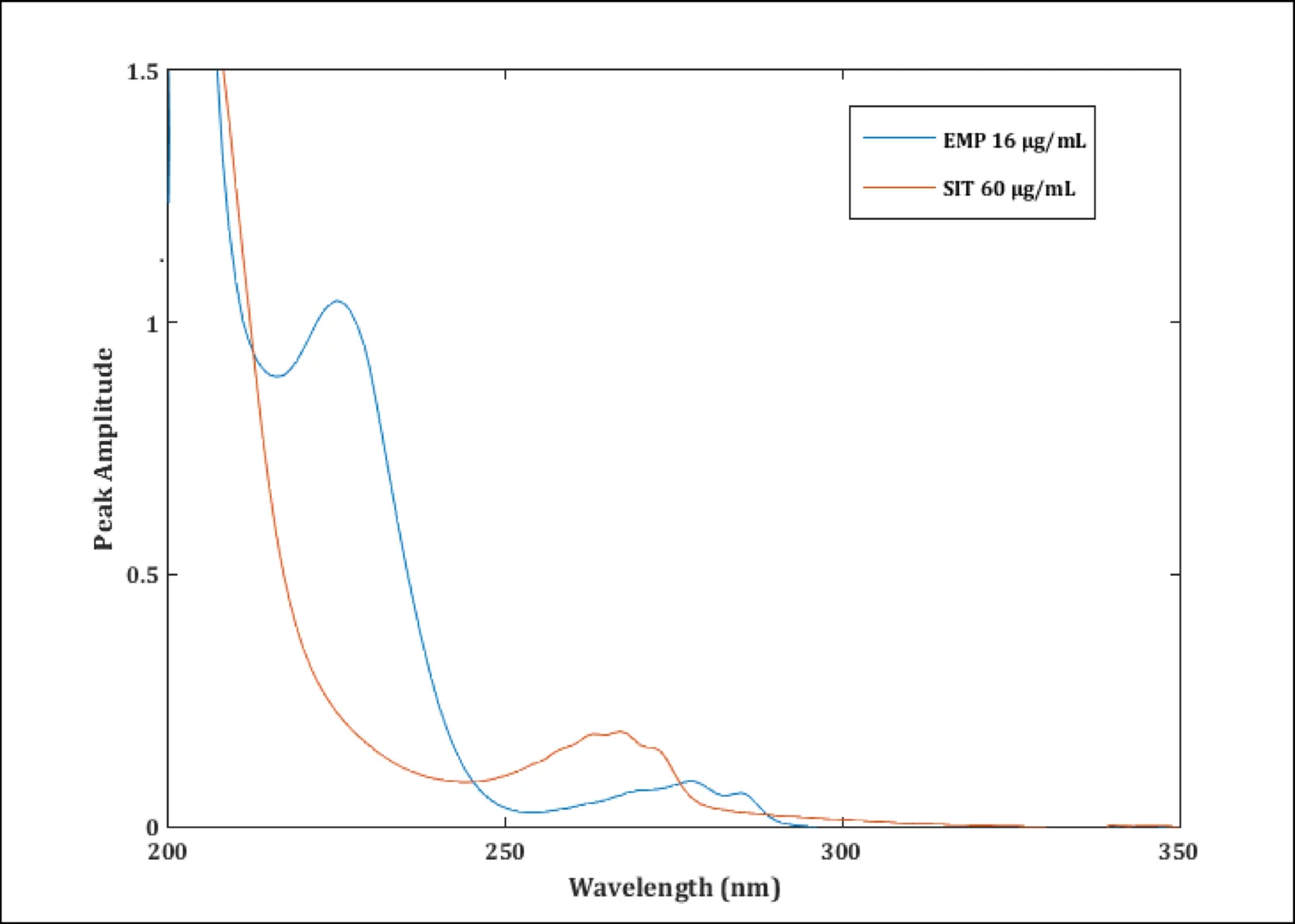
Absorption spectra of EMP and SIT

The simultaneous determination of EMP and SIT presents a considerable analytical challenge because their zero-order UV absorption spectra overlap significantly (Fig. 2). This spectral overlap prevents the reliable direct spectrophotometric determination of either drug in the presence of the other. Therefore, mathematical spectral manipulation was investigated as an effective strategy for resolving the overlapping signals without a prior physical separation step. Accordingly, two complementary ratio-spectrum methods—RD and 1DD of ratio spectrophotometry—were developed. These methods were designed to enable accurate, selective, and separation-free simultaneous determination of EMP and SIT in their combined pharmaceutical formulations.

### Optimization of the RD and 1DD spectrophotometric methods

For the ratio-spectrum procedures, the zero-order absorption spectrum of each analyte was divided by the spectrum of the other drug, which was used as the divisor. The divisor concentration is a critical parameter because it affects the shape, amplitude, smoothness, and signal-to-noise ratio of the resulting ratio spectra. Accordingly, several divisor concentrations were examined to select spectra with suitable amplitude, minimal noise, and well-defined spectral profiles for subsequent manipulation.

An EMP concentration of 18 µg/mL was selected as the divisor for generating the ratio spectra of SIT, whereas an SIT concentration of 80 µg/mL was selected as the divisor for generating the ratio spectra of EMP. At these concentrations, the obtained ratio spectra were smooth and sufficiently resolved, with appropriate signal intensity and no appreciable spectral distortion. The selected divisor concentrations were therefore used throughout the subsequent RD and 1DD procedures.

### Ratio difference method

The RD method measures the difference between the amplitudes of a ratio spectrum at two selected wavelengths. The selected wavelength pairs should provide linear responses for the target analyte while maintaining a constant contribution from the divisor component; subtraction of these amplitudes consequently eliminates the constant contribution of the interfering component.

For EMP, the difference in ratio amplitudes at 230 and 285 nm provided a selective and linear response. Accordingly, ΔP 230 − 285 was used to quantify EMP. For SIT, the amplitude difference at 257 and 209 nm.

**Fig. 3:**
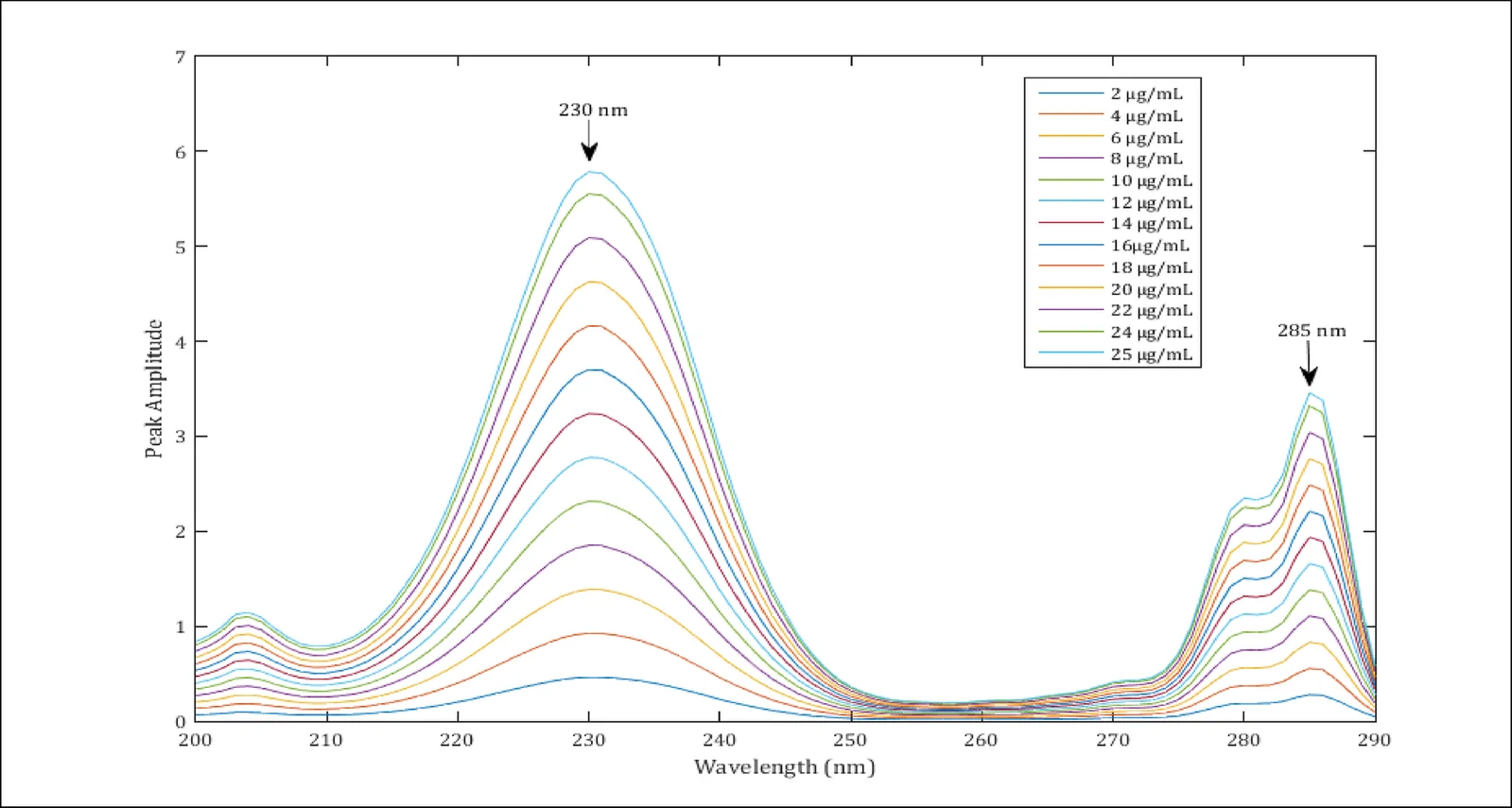
Ratio spectra of EMP utilizing an 80 mg/ml solution of SIT as a divisor

**Fig. 4:**
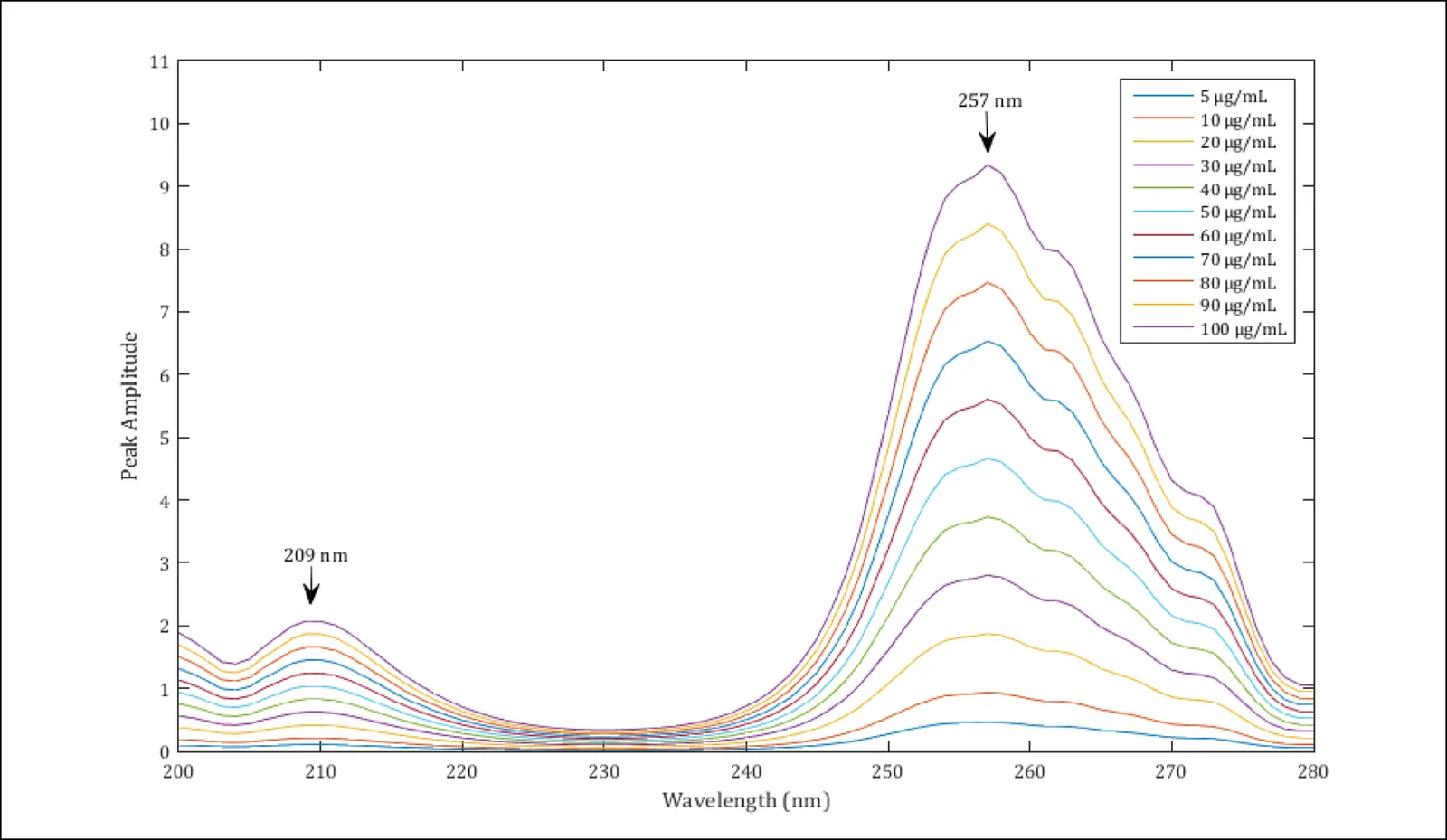
Ratio spectra of SIT utilizing an 18 mg/ml solution of EMP as a divisor

ΔP 257−209 was selected for quantitative determination. Figs. 3 and 4.

These wavelength pairs enabled selective determination of each analyte in the presence of the other without a prior physical separation step. The RD calibration plots were linear over concentration ranges of 2–25 µg/mL for EMP and 5–100 µg/mL for SIT, with coefficients of determination (^2^) of 0.9999 and 0.9997, respectively. These results demonstrate a strong proportional relationship between the measured ratio-amplitude differences and analyte concentration.

### First-derivative ratio method

1DD ratio spectrophotometry was investigated as a complementary ratio-spectrum manipulation approach for resolving the pronounced overlap between the absorption spectra of EMP and SIT. The ratio spectra were converted to their first-derivative form using a wavelength increment (Δ) of 8 nm and a scaling factor of 80. These parameters were selected to achieve adequately resolved derivative signals with suitable amplitude while limiting noise amplification.

**Fig. 5:**
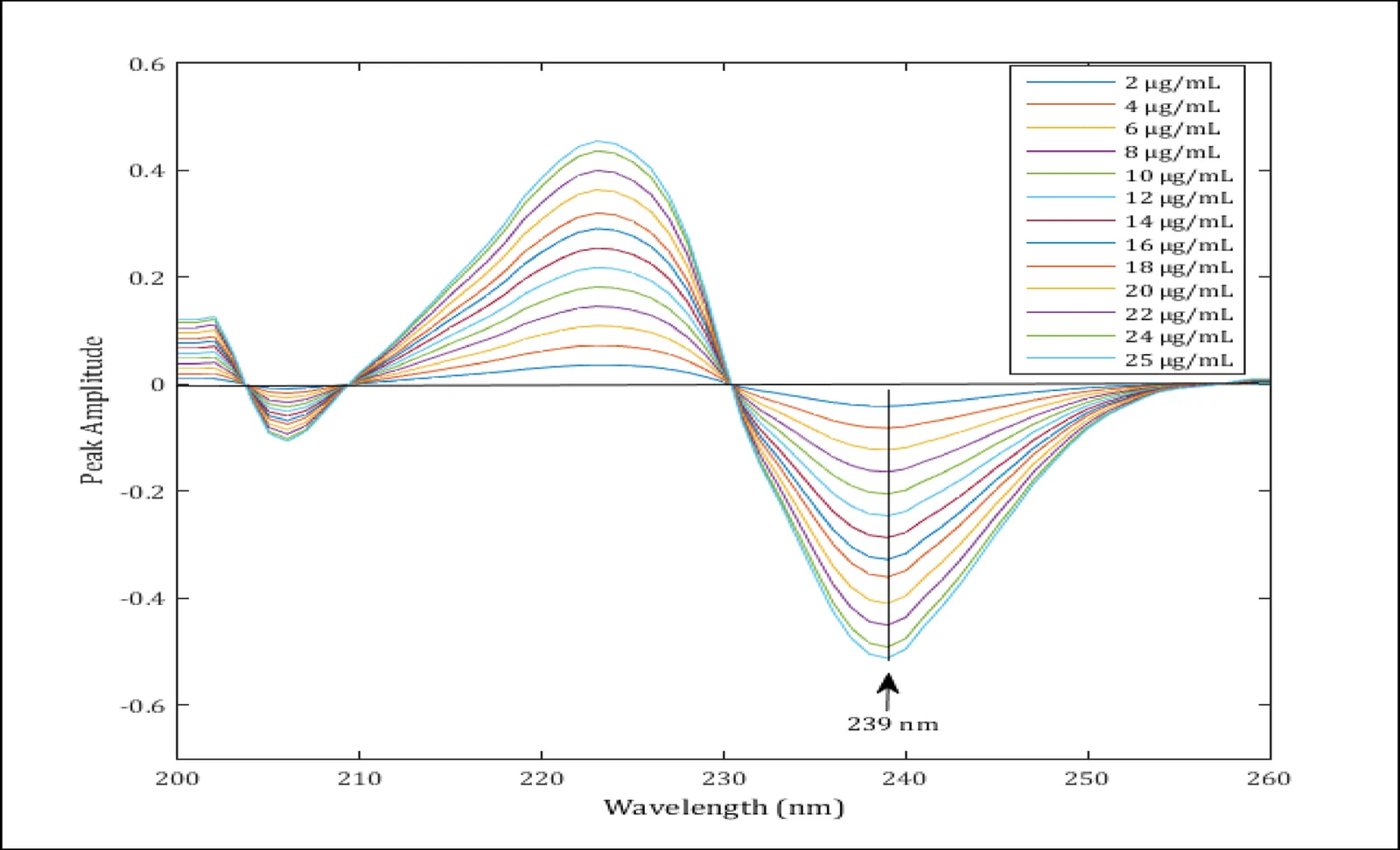
^1^DD spectra of EMP using 80 mg/ml of SIT as a divisor

**Fig. 6:**
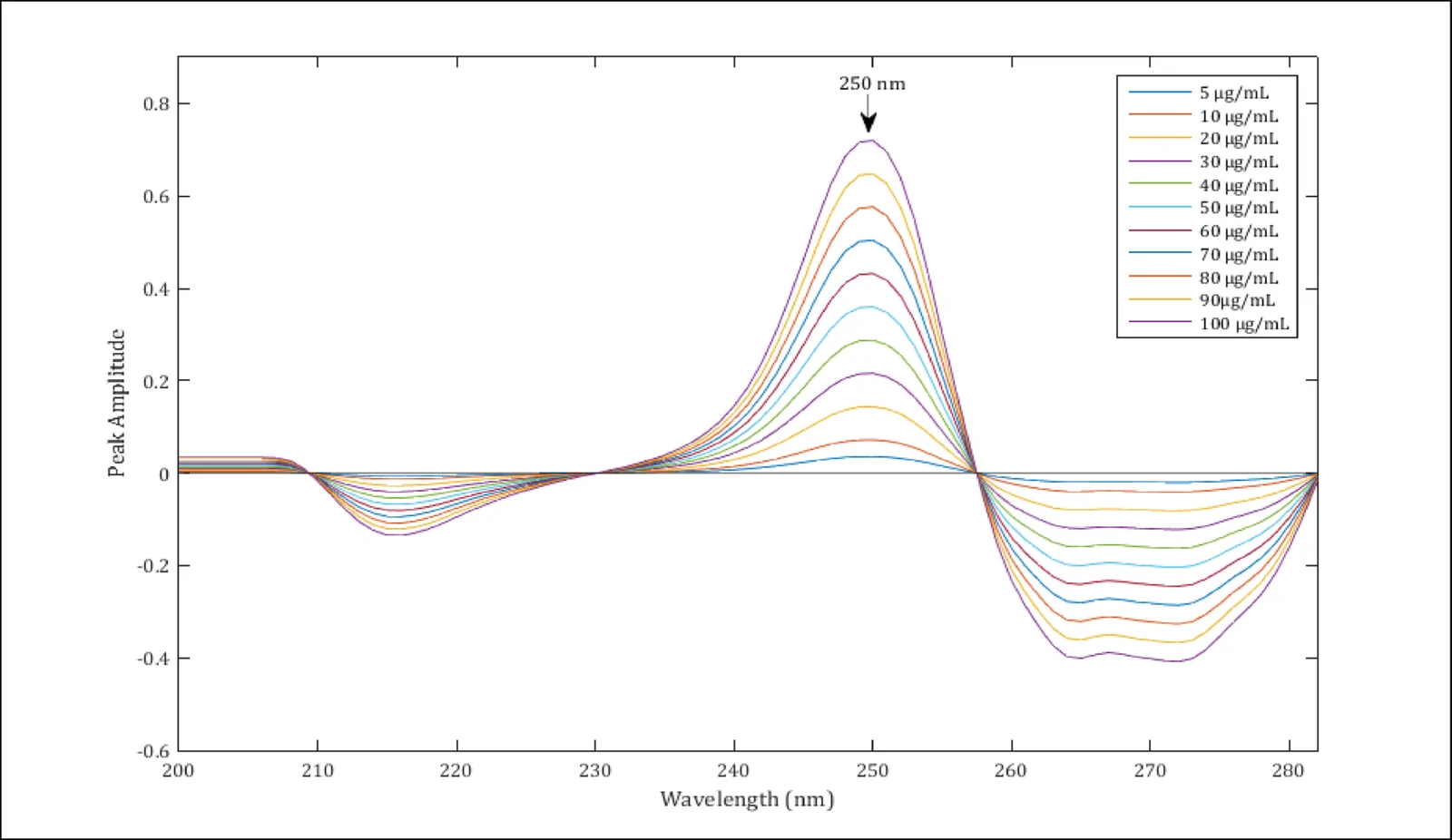
^1^DD spectra of SIT using 18 mg/ml of EMP as a divisor

The first-derivative ratio spectra were evaluated at several wavelengths, and 239 nm and 250 nm were selected for EMP and SIT, respectively, based on the linearity, signal intensity, and selectivity of the corresponding derivative responses. At these wavelengths, the contribution of the coexisting drug was negligible, (Figs. 5 and 6).

The 1DD calibration plots were linear over concentration ranges of 2–25 µg/mL for EMP and 5–100 µg/mL for SIT, with R^2^ values of 0.9998 for both analytes. These findings confirm that the 1DD procedure enables reliable mathematical resolution of the overlapping EMP and SIT spectra without requiring a physical separation step.

Overall, the 1DD method provides a complementary alternative to the RD procedure. While RD relies on differences between ratio amplitudes at two wavelengths, 1DD employs derivative transformation of the ratio spectra to generate selective analytical responses for each analyte.

### Method validation

The proposed methods were validated in accordance with relevant ICH recommendations for linearity, accuracy, precision, selectivity, robustness, and sensitivity.

#### Linearity

The RD method showed excellent linearity over the concentration ranges of 2–25 µg/mL for EMP and 5–100 µg/mL for SIT. The corresponding determination coefficients (r²) were 0.9999 and 0.9997, respectively. The regression slopes were 0.0934 and 0.0722, while the intercepts were −0.0016 and 0.0023 for EMP and SIT, respectively.

Similarly, the 1DD method exhibited excellent linearity over 2–25 µg/mL for EMP and 5–100 µg/mL for SIT, with r² values of 0.9998 for both analytes. The corresponding slopes were −0.0206 and 0.0072, while the intercepts were 0.0019 and −0.0020 for EMP and SIT, respectively (Table 1).

**Table 1:** Analytical and regression parameters obtained for the determination of EMP and SIT using the proposed RD and 1DD spectrophotometric methods

Parameters	RD		^1^DD	
	EMP	SIT	EMP	SIT
Wavelength (nm)	230–285	257 − 209	239	250
Linearity range (µg/ml)	2–25	5–100	2–25	5–100
LOD (µg/ml)	0.326	0.419	0.324	0.423
LOQ (µg/ml)	0.978	1.257	0.972	1.269
-Slope (b)	0.0934	0.0722	−0.0206	0.0072
-Intercept (a)	−0.0016	0.0023	0.0019	−0.002
Coefficient of determination (r^2^)	0.9999	0.9997	0.9998	0.9998
Accuracy (%R) ^a^	100.11	99.18	99.74	99.32
Repeatability precision (RSD)^b^	0.761	0.504	0.452	0.653
Intermediate precision (RSD)^b^	0.979	0.651	0.814	0.716

^a^Average of nine determinations (three concentrations repeated three times)

^b^RSD of nine determinations (three concentrations repeated three times)

#### Accuracy

The accuracy of the proposed procedures was evaluated using recovery studies. The RD method provided mean recoveries of 100.11% for EMP and 99.18% for SIT, whereas the corresponding recoveries obtained using the 1DD method were 99.74% and 99.32%, respectively.

#### Precision

The precision of the proposed methods was evaluated for repeatability and intermediate precision. For the RD method, repeatability %RSD values were 0.761% for EMP and 0.504% for SIT, while intermediate precision values were 0.979% and 0.651%, respectively. For the 1DD method, repeatability values were 0.452% for EMP and 0.653% for SIT, with corresponding intermediate precision values of 0.814% and 0.716% (Table 1). The consistently low %RSD values obtained using the three approaches confirm their excellent repeatability and reproducibility and demonstrate their suitability for routine quantitative analysis.

#### Sensitivity

The limits of detection (LOD) and quantification (LOQ) were calculated from the calibration-curve characteristics according to the ICH-recommended statistical approach:

$$LOD{\text{ }} = {\text{ }}3.3\sigma /S$$

$$LOQ{\text{ }} = {\text{ }}10\sigma /S$$

where S is the slope of the corresponding calibration curve, and σ is the standard deviation of the intercept obtained from the regression analysis of the calibration data. is the standard deviation of the intercept obtained from the regression analysis of the calibration data. The calculated LOD values for the RD method were 0.326 and 0.419 µg/mL for EMP and SIT, respectively, whereas those for the 1DD method were 0.324 and 0.423 µg/mL. The corresponding LOQ values were 0.978 and 1.257 µg/mL for EMP and SIT using RD, and 0.972 and 1.269 µg/mL using 1DD (Table 2).

**Table 2:** Determination of EMP and SIT in the lab—prepared mixtures using the proposed methods

Added (µg/ml)		RD				^1^DD			
		EMP		SIT		EMP		SIT	
EMP	SIT	Found	%Recovery^(a)^	Found	%Recovery^(a)^	Found	%Recovery^(a)^	Found	%Recovery^(a)^
3	12	2.96	98.67	11.83	98.58	3.01	100.33	11.9	99.17
6	24	6.02	100.33	23.94	99.75	5.98	99.67	23.81	99.21
9	36	8.92	99.11	36.08	100.22	8.96	99.56	36.05	100.14
12	46	11.85	98.75	45.79	99.54	11.91	99.25	45.87	99.72
15	60	15.07	100.47	60.23	100.38	15.12	100.80	60.1	100.17
Mean ± %RSD		99.47 ± 0.80		99.69 ± 0.67		99.92 ± 0.64		99.68 ± 0.44	

^a^Average of five measurements

#### Selectivity

The selectivity of the proposed RD and 1DD methods was further assessed to verify that the determination of EMP and SIT was not adversely affected by the tablet matrix. Because the manufacturer did not disclose the complete qualitative and quantitative composition of the commercial tablet excipients, a representative laboratory-prepared placebo matrix was used. The placebo was prepared in the absence of EMP and SIT and included the coloring agents declared on the product label, namely ferric oxide red, ferric oxide yellow, and titanium dioxide, together with commonly used non-active tablet excipients (microcrystalline cellulose, lactose monohydrate, starch, talc, and magnesium stearate).

The placebo preparation underwent the same extraction, dilution, filtration, and measurement procedures used for the commercial tablet samples. The resulting placebo solution was scanned over the investigated spectral range and subsequently processed according to the proposed RD and 1DD procedures. No detectable interfering response attributable to the tested placebo components was observed at the selected analytical wavelengths or in the corresponding ratio and first-derivative ratio spectra.

To provide additional experimental evidence of matrix selectivity, the placebo preparation was fortified with known concentrations of EMP and SIT and analyzed using the proposed methods. Recoveries ranged from 99.05 to 99.58% for EMP and 98.61–99.76% for SIT, with %RSD values not exceeding 1.21%. The close agreement between the added and recovered concentrations, together with the low variability, indicates that the tested placebo matrix did not introduce appreciable interference or systematic bias into the determination of either analyte. Collectively, the absence of a detectable placebo response and the satisfactory recoveries obtained from placebo-spiked preparations demonstrate that the tested matrix did not cause appreciable interference under the proposed analytical conditions, supporting the selectivity of the RD and 1DD methods for simultaneous determination of EMP and SIT in the investigated tablet formulation.

### Analysis of commercial tablets

The proposed RD and 1DD procedures were applied to simultaneously determine EMP and SIT in Gliempaflozin^®^ SITA 25/100^®^ tablets. The assay results closely agreed with the labeled drug contents, confirming that both methods are suitable for routine analysis of the commercial formulation.

Standard addition was subsequently performed by spiking tablet sample solutions with known amounts of EMP and SIT. Satisfactory recoveries of 99.46%–100.19% and low %RSD values of ≤ 1.05% were obtained, providing additional evidence that the tablet matrix did not adversely affect quantification (Table 3).

**Table 3:** Determination of EMP and SIT in Gliempaflozin^®^ SITA 25/100 tablets using the proposed method and results of the standard addition technique

Method	Drug	Pharmaceutical taken (µg/ml)	Pharmaceutical found^a^ (µg/ml)	Pure added (µg/ml)	Pure found^b^ (µg/ml)	Pure recovery (%*R*)
RD	EMP	25	25.2	4	4.01	100.25
				8	7.88	98.5
				16	15.79	98.69
				20	20.1	100.5
		Mean ± %RSD				99.48 ± 1.04
	SIT	100	99.73	16	15.81	98.81
				32	31.95	99.84
				64	64.31	100.48
				80	80.28	100.35
		Mean ± %RSD				99.87 ± 0.73
^1^DD	EMP	25	25.19	4	3.97	99.25
				8	7.91	98.875
				16	15.86	99.125
				20	20.12	100.6
		Mean ± %RSD				99.46 ± 0.77
	SIT	100	100.1	16	15.94	99.63
				32	32.2	100.63
				64	64.16	100.25
				80	80.22	100.28
		Mean ± %RSD				100.19 ± 0.42

^a^Average of five determinations

^b^Average of three determinations

### Statistical comparison with the reported method

To further establish the validity of the proposed procedures, the assay results obtained using the RD and 1DD methods were statistically compared with those obtained using the reported method for the same commercial formulation. Student’s *t*-test was used to compare the mean assay values, whereas the *F*-test was applied to compare the variances at the 95% confidence level (Table 4).

**Table 4:** Statistical comparison of the outcomes of the suggested UV-spectroscopic method and the reported method for determining EMP and SIT

Parameters	EMP			SIT		
	RD	^1^DD	Reported method	RD	^1^DD	Reported method
%R	100.36 ^a^	99.48 ^a^	100.17 ^a^	100.23 ^a^	99.81 ^a^	100.49 ^a^
SD	0.77	1.29	1.12	0.97	0.64	1.32
Variance	0.59	1.66	1.25	0.94	0.41	1.74
Student’s *t-test* ^*b*^ (2.306)	0.313	0.905	—	0.355	1.04	—
*F-value* ^*b*^ *(6.388)*	2.12	1.33	—	1.85	4.24	—

^a^Average of five determinations

^b^The values in parentheses are tabulated values of t and F at(P=0.05)

For EMP, the calculated *t* values for RD and 1DD were 0.581 and 0.753, respectively, compared with the tabulated value of 2.306. The corresponding calculated *F* values were 2.12 and 1.33, compared with the tabulated value of 6.388. For SIT, the calculated *t* values were 0.612 and 0.796, while the corresponding *F* values were 1.85 and 4.24. All calculated *t* and *F* values were lower than their respective critical values, indicating no statistically significant differences between the proposed and reported methods at the 95% confidence level.

These findings support the analytical equivalence of the proposed spectrophotometric procedures to the reported method while offering the operational advantages of direct UV measurement without chromatographic separation.

### Comparison with previously reported methods

Several analytical procedures have been reported for the simultaneous determination of EMP and SIT, including simultaneous-equation UV spectrophotometry and RP-HPLC [53, 54]. These approaches provide established analytical solutions; however, differences in spectral treatment, working concentration ranges, instrumentation, and operational requirements are relevant when selecting a method for routine analysis of combined pharmaceutical formulations.

A previously reported simultaneous-equation UV spectrophotometric method was developed to determine EMP and SIT in bulk form [53]. The method used methanol (5:5, v/v) as the solvent and absorption maxima at 267 nm for SIT and 276.2 nm for EMP. Both analytes exhibited linear responses over the concentration range of 50–250 µg/mL, with coefficients of determination of 0.9957 for SIT and 0.9981 for EMP. The method provided satisfactory accuracy and precision with a simple and rapid analytical procedure. Simultaneous-equation spectrophotometry offers the advantage of direct quantitative determination without a separation step; however, the calculation is based on the absorptivity coefficients of the individual analytes at selected wavelengths. Accordingly, its quantitative performance depends on the spectral characteristics of the components and the suitability of the selected wavelength pair, particularly when substantial spectral overlap is present.

The present methods approach the same analytical challenge from a different spectral-processing perspective. The RD method utilizes ratio spectra and quantifies each analyte from the difference between amplitudes measured at two selected wavelengths, whereas the 1DD method combines ratio-spectrum manipulation with first-derivative processing to provide additional spectral discrimination. These approaches reduce the contribution of one component while monitoring the other’s response, enabling simultaneous determination without physical separation. In addition, the proposed procedures were optimized for analysis of the combined pharmaceutical formulation at substantially lower working concentrations, covering 2–25 µg/mL for EMP and 5–100 µg/mL for SIT following appropriate sample dilution. This working range extends the concentration domain applicable to formulation analysis toward lower levels than those reported for the simultaneous-equation procedure.

This distinction is relevant to the analysis of marketed combined formulations, where reliable individual responses are required in the presence of strongly overlapping spectra. The proposed RD and 1DD procedures achieve this through complementary mathematical treatments while retaining the inherent advantages of direct UV–visible analysis. The use of ethanol as the analytical solvent further supports a straightforward sample-preparation procedure based on dissolution and dilution, without chromatographic separation or chemical derivatization.

The reported RP-HPLC method provides an alternative based on physical separation and was developed for the simultaneous determination of EMP and SIT in pharmaceutical formulations [54]. Using a C18 column and a methanol: 0.05% acetic acid (45:55, v/v) mobile phase, the method provided linearity over 50–150 µg/mL for both analytes, with retention times of approximately 2.75 min for SIT and 3.77 min for EMP. HPLC provides high selectivity through physical separation of the analytes and is well suited to applications requiring chromatographic resolution. However, implementation requires an HPLC system, analytical column, mobile-phase preparation, controlled flow conditions, and routine column maintenance. These requirements generally involve greater instrumentation, mobile-phase, and operational demands than direct UV–visible spectrophotometry.

Accordingly, the proposed RD and 1DD procedures provide complementary alternatives to the available analytical approaches for EMP and SIT. Rather than relying on physical chromatographic separation, they exploit UV spectral information to discriminate between the two overlapping components. RD provides a simple ratio-based quantitative approach, whereas 1DD offers an additional level of spectral discrimination through derivative processing. Their applicability to the combined tablet formulation, relatively low working concentrations, ethanol-based sample preparation, and compatibility with conventional UV–visible instrumentation collectively support their practical use in routine pharmaceutical quality control (Table 5).

**Table 5:** Comparative analytical characteristics of the proposed RD and 1DD methods and previously reported methods for simultaneous determination of empagliflozin and sitagliptin

Parameter	Reported UV Method [53]	Reported RP-HPLC [54]	Proposed RD	Proposed 1DD
EMP linear range µg/mL	50–250	50–150	2–25	2–25
SIT linear range µg/mL	50–250	50–150	5–100	5–100
Analysis time	< 3 min	< 5 min	< 3 min	< 3 min
Solvent/mobile phase	Methanol: water (5:5, v/v)	Methanol:0.05% acetic acid (45:55, v/v)	Ethanol	Ethanol
Solvent safety/environmental burden	Moderate	Higher due to mobile-phase consumption and chromatographic waste	Lower ethanol-based	Lower ethanol-based
Physical separation	Not required	Required	Not required	Not required
Sample preparation	Dilution in methanol: water	Extraction/dilution followed by chromatographic injection	Simple dissolution/dilution in ethanol	Simple dissolution/ dilution in ethanol
Relative operational cost	Low	Higher	Very low	Very low
Instrument Requirement	UV–Vis spectrophotometer	HPLC system and column	UV–Vis spectrophotometer	UV–Vis spectrophotometer
Application	Bulk drug	Pharmaceutical formulations	Pharmaceutical formulations	Pharmaceutical formulations

Overall, the proposed procedures extend the available analytical options for the EMP/SIT combination by applying complementary ratio-based spectrophotometric strategies to combined pharmaceutical formulations. They provide an alternative to both direct simultaneous-equation calculations and chromatographic separation while maintaining a simple, accessible, and separation-free analytical workflow. Their analytical performance and practical characteristics, together with the subsequent sustainability assessment, support their use as complementary UV–visible methods for routine determination of EMP and SIT in combined pharmaceutical products.

### Greenness, whiteness, and practical applicability assessment

To provide a comprehensive, quantitative, and multidimensional assessment of the proposed analytical procedures, five complementary evaluation tools were applied: the Carbon Footprint Reduction Index (CaFRI), ComplexMoGAPI, Click Analytical Chemistry Index (CACI), Blue Applicability Grade Index (BAGI), and Whiteness Evaluation for Chemical Analysis (WECA). The proposed RD and 1DD procedures were benchmarked against the previously reported simultaneous-equation spectrophotometric method for the simultaneous determination of EMP and SIT [53] (Table 6). The individual metrics were considered according to their respective evaluation domains, encompassing carbon footprint, environmental impact, resource consumption, practical applicability, and the overall balance between analytical performance and sustainability. The resulting profiles provide a multidimensional comparison of the proposed and reported procedures and highlight their respective environmental and practical characteristics.

**Table 6:**
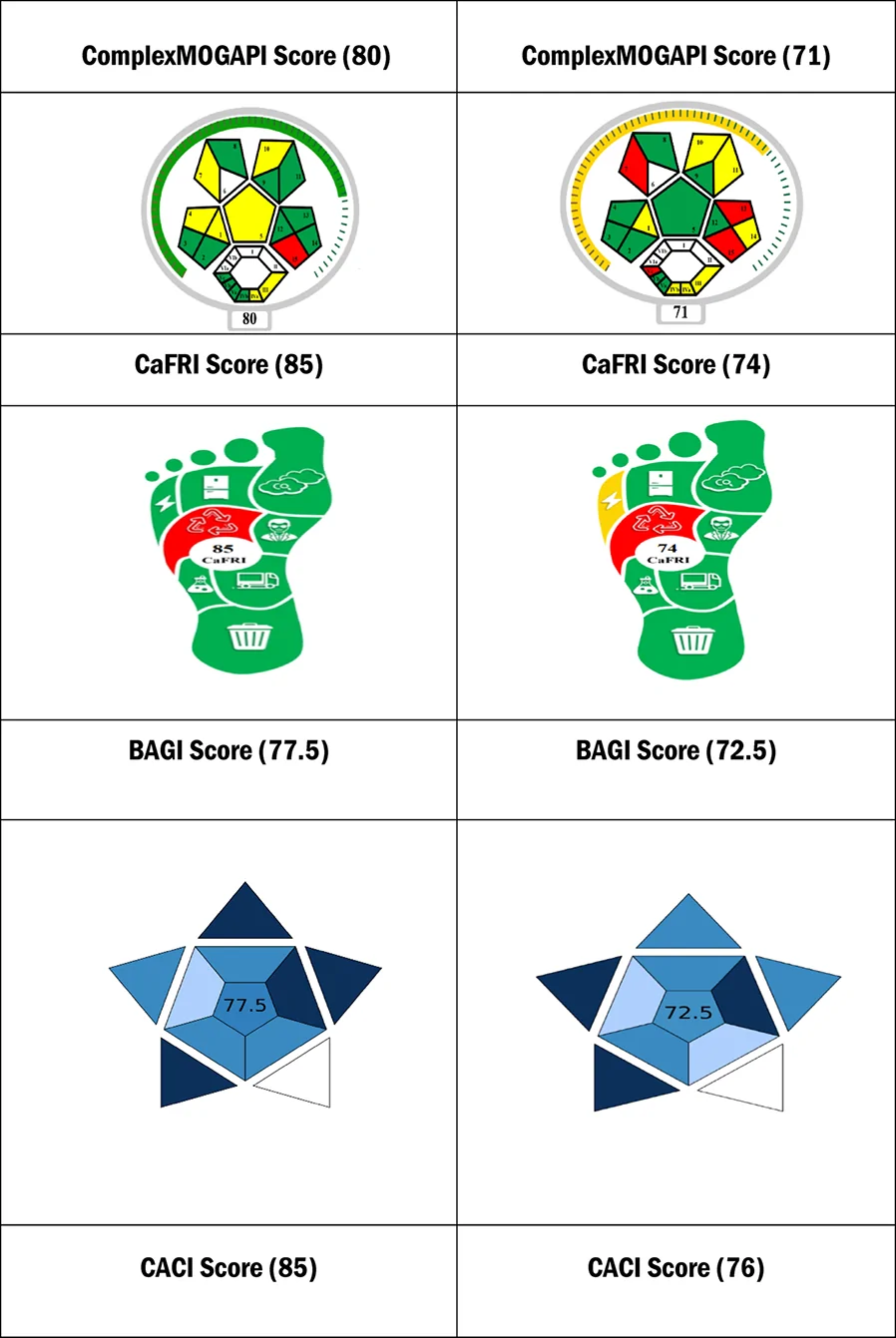

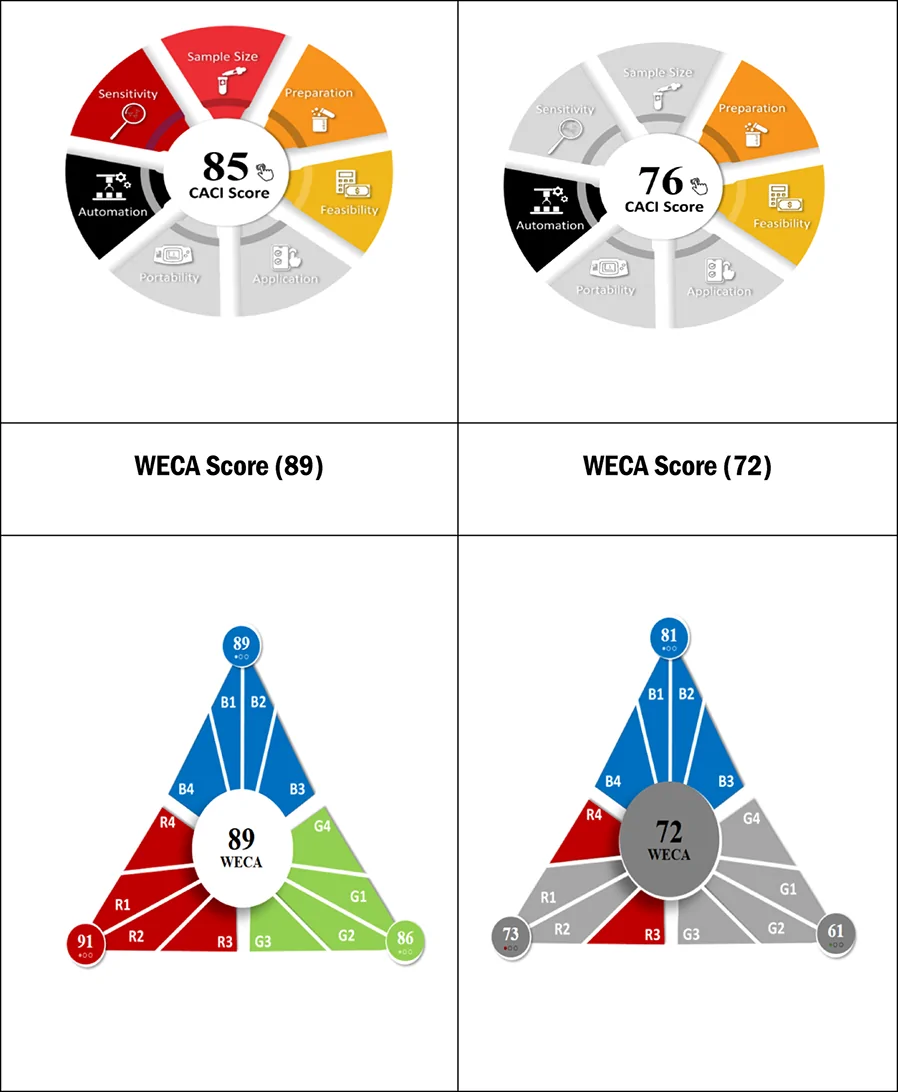
Greenness and blueness assessment of the proposed methods

#### Carbon footprint reduction index (CaFRI)

The proposed RD and 1DD methods achieved a CaFRI score of 85, compared with 74 for the reported simultaneous-equation method. The 11-point higher score indicates a more favorable carbon-footprint profile for the proposed analytical workflow under the assessment conditions. CaFRI evaluates the carbon-footprint implications of the analytical procedure as a whole; therefore, the difference between the two procedures should not be attributed to solvent selection alone.

The favorable CaFRI score of the proposed methods is consistent with their relatively low-resource workflow, which involves sample dissolution, dilution, direct UV–visible measurement, and mathematical data processing. The procedures do not require chromatographic separation, stationary phases, continuous mobile-phase delivery, or separation-associated consumables. In addition, the proposed methods use ethanol as the analytical solvent, whereas the reported simultaneous-equation method employs methanol (5:5, v/v). Accordingly, the higher CaFRI score reflects the combined characteristics of the complete analytical workflow, including solvent requirements and associated resource consumption, rather than the solvent’s environmental characteristics in isolation.

#### ComplexMoGAPI

The proposed methods obtained a ComplexMoGAPI score of 80, compared with 71 for the reported simultaneous-equation method. The higher score indicates a more favorable profile within this multidimensional assessment. ComplexMoGAPI considers multiple characteristics of an analytical procedure, including solvent and reagent requirements, sample preparation, waste generation, and other operational aspects; thus, it provides a broader evaluation than metrics focused predominantly on a single environmental dimension.

The score obtained by the proposed methods is consistent with their simplified analytical workflow. Sample treatment involves dissolution and dilution followed by direct spectrophotometric measurement, without derivatization, preconcentration, or physical separation. Ratio-spectrum manipulation provides the mathematical discrimination needed to determine EMP and SIT simultaneously without an additional separation step. Although the reported simultaneous-equation method is also separation-free, the proposed procedures combine this advantage with an ethanol-based solvent system and ratio-based spectral processing. The 80 versus 71 scores therefore indicate a more favorable multidimensional sustainability profile for the proposed workflow under the evaluated conditions.

#### Click analytical chemistry index (CACI)

The proposed RD and 1DD methods achieved a CACI score of 85, compared with 76 for the reported simultaneous-equation method, representing a 9-point higher score for the proposed procedures. CACI provides an additional carbon- and resource-oriented perspective and therefore complements the broader multidimensional assessment provided by ComplexMoGAPI.

The higher CACI score reflects the proposed workflow’s limited resource requirements. Direct UV–visible analysis eliminates separation-related operations and avoids chromatographic columns and continuous mobile-phase consumption. In addition, the proposed procedures employ a straightforward dissolution–dilution sample preparation without preconcentration or derivatization. Collectively, these characteristics contribute to the favorable carbon- and resource-related profile reflected by the CACI assessment.

#### Blue applicability grade index (BAGI)

Unlike CaFRI, ComplexMoGAPI, and CACI, which primarily address environmental and resource-related characteristics, BAGI specifically evaluates the practical applicability of an analytical procedure. The proposed methods achieved a BAGI score of 77.5, compared with 72.5 for the reported simultaneous-equation method. The 5-point higher score indicates a more favorable practical applicability profile for the proposed procedures.

The BAGI assessment considers practical attributes such as the analytical format, number of simultaneously determined analytes, instrumentation requirements, sample preparation, throughput, reagent and material requirements, need for preconcentration, degree of automation, and sample consumption. The score of 77.5 therefore reflects the favorable operational characteristics of the proposed procedures, including the use of conventional UV–visible instrumentation, straightforward sample preparation, simultaneous determination of two analytes, absence of preconcentration, and direct analysis without chromatographic separation. These features are particularly relevant to routine pharmaceutical quality-control laboratories, where accessible instrumentation, uncomplicated sample handling, and limited operational requirements are advantageous.

Although the reported simultaneous-equation procedure also benefits from direct and separation-free UV analysis, the proposed RD and 1DD methods provide complementary spectral-processing strategies for resolving the pronounced spectral overlap between EMP and SIT. RD uses amplitude differences in ratio spectra, whereas 1DD combines ratio-spectrum manipulation with derivative processing to provide additional spectral discrimination. Importantly, the BAGI score represents practical applicability rather than analytical accuracy, sensitivity, or selectivity; these analytical characteristics were evaluated independently during method validation.

#### Whiteness evaluation for chemical analysis (WECA)

To complement the separate environmental and practical assessments, the proposed methods and the reported simultaneous-equation method were also evaluated using the Whiteness Evaluation for Chemical Analysis (WECA) tool. WECA provides a holistic assessment within the White Analytical Chemistry framework by integrating three major dimensions: Red, representing analytical performance; Green, representing environmental sustainability; and Blue, representing practical and economic efficiency. The resulting composite score therefore provides an overall indication of the balance achieved among analytical quality, environmental responsibility, and practical usability.

The proposed RD and 1DD methods achieved a WECA score of 89, whereas the reported simultaneous-equation method obtained a score of 72. The proposed score falls within the high-performance range of the WECA framework, whereas the reported procedure falls within the intermediate range. Thus, the proposed method’s higher WECA score indicates a more favorable overall balance among the three dimensions evaluated by the tool.

The higher WECA score of the proposed procedures is consistent with their combination of validated analytical performance, favorable environmental characteristics, and practical simplicity. The proposed methods provide accurate, precise simultaneous determination of EMP and SIT over practically relevant concentration ranges using a separation-free UV–visible workflow with ethanol-based sample preparation. The resulting balance is reflected in the high WECA score and complements the more focused information provided by CaFRI, ComplexMoGAPI, CACI, and BAGI. Importantly, interpret WECA as an integrated whiteness assessment rather than an independent replacement for individual analytical validation, greenness, or applicability assessments.

#### Integrated assessment of the proposed and reported procedures

The five complementary metrics consistently favored the proposed RD and 1DD methods over the reported simultaneous-equation procedure. The proposed methods achieved scores of 85 versus 74 for CaFRI, 80 versus 71 for ComplexMoGAPI, 85 versus 76 for CACI, 77.5 versus 72.5 for BAGI, and 89 versus 72 for WECA, respectively. Collectively, these results indicate more favorable performance of the proposed procedures across the evaluated dimensions of sustainability, resource efficiency, practical applicability, and analytical whiteness.

Both the proposed and reported procedures are separation-free UV spectrophotometric approaches; therefore, their comparative profiles reflect differences in the overall analytical workflow rather than simply the presence or absence of a separation step. Relevant factors include the solvent system, material and resource requirements, sample handling, waste-generating operations, spectral-processing strategy, and practical implementation.

The proposed procedures employ ethanol-based sample preparation with limited solvent and reagent requirements, simple dissolution–dilution treatment, and no preconcentration or physical separation. They are also compatible with conventional UV–visible instrumentation. In addition, ratio-based spectral processing provides the required discrimination between the strongly overlapping EMP and SIT spectra without introducing additional chemical or instrumental operations. These features are consistent with the favorable profiles obtained from the individual assessment tools.

Overall, the combined results of CaFRI, ComplexMoGAPI, CACI, BAGI, and WECA provide a multidimensional characterization of the proposed RD and 1DD procedures in terms of environmental sustainability, carbon and resource efficiency, practical applicability, and analytical whiteness. The concordant outcomes across the five complementary metrics indicate that the proposed procedures offer lower-resource and practically applicable alternatives to the reported simultaneous-equation method for routine quality-control determination of EMP and SIT in combined pharmaceutical formulations.

## Conclusion

Two ratio-manipulating spectrophotometric procedures, namely Ratio Difference (RD) and first-derivative ratio (1DD) spectrophotometry, were successfully developed and validated for the simultaneous determination of EMP and SIT in their combined tablet formulation. Both procedures effectively addressed the pronounced spectral overlap between the two drugs without chromatographic separation, complex extraction, or extensive sample preparation. They exhibited satisfactory linearity, accuracy, precision, sensitivity, and robustness over their respective working ranges, demonstrating their suitability for quantitative pharmaceutical analysis.

Successful application to a commercial fixed-dose formulation, together with the absence of statistically significant differences from the reported reference method, further demonstrated the analytical validity of the proposed approaches for routine quality-control analysis. The two procedures provide complementary analytical options: RD offers a simple and direct approach based on amplitude differences in ratio spectra, whereas 1DD provides additional spectral discrimination through derivative processing. Both procedures can be implemented using conventional UV–visible spectrophotometric instrumentation without chromatographic separation.

The sustainability and practical-applicability assessments showed favorable profiles for the proposed procedures. The use of ethanol as the principal solvent, limited solvent and reagent consumption, simplified sample preparation, and reliance on conventional instrumentation contribute to reduced resource consumption and operational burden. The combined application of CaFRI, ComplexMoGAPI, CACI, BAGI, and WECA provided complementary information on environmental sustainability, carbon and resource efficiency, practical applicability, and analytical whiteness.

Overall, the proposed RD and 1DD procedures provide simple, economical, separation-free, and lower-resource alternatives for the simultaneous quality-control determination of EMP and SIT in combined pharmaceutical formulations. Their combination of adequate analytical performance, straightforward operation, and favorable sustainability characteristics makes them suitable for routine pharmaceutical quality-control applications.

The sustainability and practical applicability assessments further demonstrated the favorable profile of the proposed methods. Their use of ethanol as the principal solvent, limited solvent and reagent consumption, simplified sample preparation, and reliance on conventional instrumentation contribute to reducing operational and environmental burdens. The complementary application of CaFRI, ComplexMoGAPI, CACI, and BAGI provided a multidimensional evaluation of both environmental sustainability and practical applicability, with the BAGI score of 77.5 indicating favorable suitability for routine implementation.

Overall, the proposed RD and 1DD methods provide simple, economical, separation-free, and lower-resource alternatives for the simultaneous quality-control determination of EMP and SIT in combined pharmaceutical formulations. Their combination of adequate analytical performance, straightforward operation, and favorable sustainability characteristics makes them particularly attractive for routine pharmaceutical quality-control laboratories.

## Data Availability

The data and materials used in this study are available on reasonable request.
